# Complications of therapeutic plasma exchange in patients with neurological disorders

**Published:** 2020-01-05

**Authors:** Mahdieh Afzali, Shahram Oveisgharan, Sahebeh Rajabkhah, Siamak Abdi

**Affiliations:** 1 Department of Neurology, School of Medicine, Isfahan University of Medical Sciences, Isfahan, Iran; 2 Rush Alzheimer’s Disease Center, Rush University Medical Center, Chicago, IL, USA; 3 Department of Neurology, Shariati Hospital, Tehran University of Medical Sciences, Tehran, Iran

**Keywords:** Plasma Exchange, Plasmapheresis, Nervous System Diseases

## Abstract

**Background:** Therapeutic plasma exchange (TPE) is the treatment of choice for many neurologic disorders. The safety of this procedure is a major concern for physicians. The aim of this study was to determine the complications of TPE in patients with neurologic disorders at a tertiary referral hospital.

**Methods:** This retrospective cross-sectional study evaluated patients with various neurologic disorders receiving TPE in neurology department of Shariati Hospital, Tehran, Iran. Major and minor complications related to TPE were recorded.

**Results:** Clinical information records of 417 TPE sessions (88 patients) were available. Mean age of patients was 40.0 ± 15.8 years. Underlying diseases included central demyelinating disorders, myasthenia gravis (MG), chronic neuropathy, Guillain-Barre syndrome (GBS), and autoimmune encephalopathy in 34.1%, 33.0%, 17.0%, 14.8%, and 1.1% of patients, respectively. Major complications occurred in 15.9% of patients and 37.5% of patients accounted for minor complications. Among major adverse effects, thrombosis, infection, and life-threatening complications were seen more commonly in patients with central vascular access (P = 0.005, P = 0.003, and P = 0.010, respectively).

**Conclusion:** TPE complications were seen more commonly in patients with central vascular access. Therefore, use of peripheral vascular access and vigilant patient monitoring by trained health providers can reduce its complications.

## Introduction

Therapeutic plasma exchange (TPE) is an extracorporeal blood purification technique that removes inflammatory mediators and antibodies which are pathogenic in numerous diseases and is used commonly in many autoimmune disorders.^1^

In Asia and Australia, TPE is most commonly used for treatment of digestive system diseases, whereas in Europe and United States of America (USA), neurologic disorders are dominant. While first experiences with TPE relate to acute life-threatening conditions, such as treatment of Guillain-Barre syndrome (GBS) or myasthenic crisis, therapeutic success has also been shown for chronic diseases where immunosuppressive therapy is often required for long-term management.^2^

Despite many benefits, TPE has some complications including complications related to replacement of fluids, cardiovascular instability, vascular access, depletion of plasma constituents, and allergic reactions.^3^^-^^5^ Several studies have evaluated these complications; however, no published articles are available in Iran. The objective of this study was to determine the complications of TPE and its predisposing factors in Iran.

## Materials and Methods

In this retrospective cross-sectional study, clinical information records of patients with neurological disorders undergoing TPE at Shariati Hospital, Tehran, Iran, from June 2015 to March 2016 were assessed.

The study was approved by the Ethics Committee of Tehran University of Medical Sciences, Tehran. Plasma exchange sessions were performed by intermittent-flow centrifuge (Hemonetics PCS2) cell separator. 2.5 liter plasma was exchanged with normal saline and albumin in each session [no other replacement fluid, e.g., fresh frozen plasma (FFP) was used, calcium supplement was used in all patients].

Patients with unavailable clinical records or missing data were excluded from the study. All adverse effects were classified in two general categories of major and minor complications. Major complications were defined as shock, pneumothorax, thrombosis [pulmonary thromboembolism (PTE), deep vein thrombosis (DVT), access site thrombosis, and sepsis]. Other adverse events were considered as minor complications. 

To compare categorical variables across strata, we used chi-square test and Fisher’s exact test when was appropriate. Comparison of continuous variables across two groups was accomplished by t-test. Mann-Whitney U test was used when the continuous variable did not have a normal distribution. The level of statistical significance was defined as P < 0.05.

## Results

From June 2015 to March 2016, TPE was performed on 110 patients, of which, data of 88 patients were recorded completely. A total of 417 TPE sessions were available. 47 patients (53.4%) were men and 41 patients (46.6%) were women. Their mean age was 40.0 ± 15.8 years ranging from 15 to 84 years.

Central demyelinating disorders accounted for 34.1% of patients followed by myasthenia gravis (MG) 33.0%, chronic neuropathy 17.0%, GBS 14.8%, and autoimmune encephalopathy 1.1% (Table 1). Plasma exchange was performed via central vascular access in 43 patients (48.9%) and peripheral vascular access was used in the remainder of 45 ones (51.1%). 

Overall complications were found in 53.4% of patients: 15.9% of which were major, including shock, pneumothorax, thrombosis, and sepsis. Patients with these systemic complications responded to intensive care unit (ICU) admission and plasmapheresis was continued. Citrate reaction (paresthesia, muscle cramp and twitch, hypocalcemia) was the most frequent complication which was observed in 15 TPE sessions in 15 patients. Most of minor events were transient and resolved with no treatments. Unfortunately, one patient died during hospitalization due to septic shock which was not attributed directly to plasmapheresis.

**Table 1:** Frequency of patients and therapeutic plasma exchange (TPE) sessions

**Diseases treated**	**Number of ** **patients**	**Treatment session ** **per patient ** **(range)**	**Total number of ** **treatment ** **session**	**Average amount of ** **fluid removed per ** **treatment (ml)**
Central demyelinating disorders	43	3-5	209	2.5
MG	29	2-5	133	2.5
Chronic neuropathy	15	3-5	70	2.4
GBS	13	4-6	64	2.5
Autoimmune encephalitis	1	5	5	2.5
Total	88	2-6	417	2.5

MG: Myasthenia gravis; GBS: Guillain-Barre syndrome

**Table 2:** Frequency of therapeutic plasma exchange (TPE) complications

**Complications**		**Patients (n = 88)**	**Procedures (n = 417)**
		**n (%)**	**n (%)**
Major	Shock	1 (1.1)	1 (0.2)
	Pneumothorax	1 (1.1)	1 (0.2)
	Thrombosis (PTE, DVT, central access site thrombosis)	7 (8.0)	8 (1.9)
	Sepsis	5 (5.7)	5 (1.2)
	Total major complications	14 (15.9)	15 (3.5)
Minor	Peripheral access site problems (access site infection, phlebitis, access site hematoma)	6 (6.7)	6 (1.4)
	Bleeding (epistaxis, menorrhagia, access site hemorrhage)	2 (2.3)	4 (1.0)
	TPE-related hypotensive symptoms (presyncope/syncope, chest discomfort, palpitation)	6 (6.8)	8 (1.9)
	Citrate reactions (paresthesia, muscle cramp, muscle twitch, hypocalcemia)	15 (17.0)	15 (3.6)
	Pneumonia	4 (4.5)	4 (1.0)
	Total minor complications	33 (37.5)	37 (8.9)
	Total	47 (53.4)	52 (12.4)

PTE: Pulmonary thromboembolism; DVT: Deep venous thrombosis; TPE: Therapeutic plasma exchange

10 patients developed complications related to vascular access, including 4 cases of access site thrombosis, 4 cases of catheter-related infection, 1 case of hemorrhage after catheter removal, and 1 case of pneumothorax related to subclavian catheter placement. Phlebitis and access site hematoma were technical complications occurring in 2 patients. No death or allergic reactions were reported during the study period (Table 2). Venous thromboembolism (VTE) prophylaxis with enoxaparin was used in 17 patients during hospitalization, among whom, one venous thrombosis occurred in comparison with 6 cases of venous thrombosis in those not receiving enoxaparin. However, the difference was not statistically significant (P > 0.999).

International normalized ratio (INR) and platelet count were not statistically different between those with and without thrombosis (P = 0.720 and P = 0.560, respectively). Moreover, infection was more common in patients getting immunosuppressive drugs (P = 0.010).

**Figure 1:**
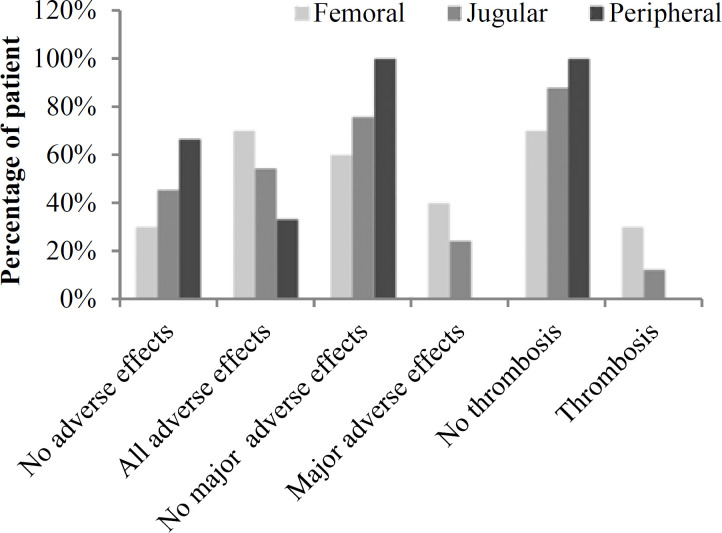
Frequency of adverse effects categorized by venous access

Frequency of thrombosis, infection, major complications, and all adverse effects was more in patients with central venous catheter (CVC) (7%, 11%, 12%, and 25%, respectively), compared to patients with peripheral venous catheter (PVC) (0%, 1%, 0%, and 15%, respectively) (P = 0.005, P = 0.003, P = 0.010, and P = 0.030) (Figure 1).

## Discussion

TPE is an extracorporeal technique that removes plasma proteins, including inflammatory mediators and antibodies, replacing them with other fluids such as albumin, FFP, or another crystalloid or colloid substance depending on patient's clinical condition. Replacing plasma with other fluids is potentially dangerous which can result in coagulation, immunity, electrolyte and drug level impairment.^5^ Moreover, hemodynamic instability and technical issues related to vascular access including catheter thrombosis, infection, or pneumothorax can complicate this procedure.

Safety is a great concern in any medical procedure. Among 88 patients with 417 TPE sessions, complications were detected in 47 patients (12.4% of sessions, 53.4% of patients). Major complications occurred in 14 sessions (3.5% of sessions, 15.9% of patients) and minor complications were seen in 33 sessions (8.9% of sessions, 37.5% of patients). Major complications consisted of one patient with shock, one patient with pneumothorax, 7 patients with thrombotic complications, and 5 patients with sepsis. Major complication occurrence in 3.5% of sessions was in accordance with previous studies which reported 0.025% to 4.75% life-threatening (major) complications per session.^6^^-^^8^

In a study, kaya et al. reported a total of 18.3% complications in 115 patients with 771 TPE sessions. Complications included those related to catheter placement procedure (8.7%), hypotension (3.5%), hypocalcemia (3.5%), allergic reaction (1.7%), and tachycardia (0.9%).^9^ Szczeklik et al. in another study on 54 ICU patients with 370 TPE sessions recorded 11.1% complication rate in all procedures, of which 2.16% were life-threatening.^10^ Gafoor et al. reported thrombophlebitis in 8.7%, DVT in 1.7%, pneumothorax in 0.9%, paresthesia or cramp in 36.1%, mild hypotension in 32.2%, moderate or severe hypotension in 5.2%, and allergy in 2.2% among 230 neurologic patients during 979 TPE sessions.^11^

Most of these studies show lower complication rate than our study. However, major complications are the same. This difference could be because of different definition of complication in different studies; as Gafoor et al. showed similar results by including mild adverse effects in their study.^11^ Other contributing factors could be using different study methodologies (antegrade vs. retrograde), different plasma exchange methods (centrifugation vs. filtration), different replacement fluids, venous access, and different patient characteristics.^8^^-^^10^^,^^12^^,^^13^

Zollner et al. showed that although TPE could cause hypofibrinogenemia, bleeding was rare in patient undergoing TPE.^14^ Our study also showed that despite the changes in the coagulation system, bleeding was not common in patients undergoing TPE and only 4 cases of minor catheter site hematoma or bleeding were seen. There was no relationship between hemorrhagic complications and baseline platelet count and INR.

In addition to the elimination of pathogens, immunoglobulin (Ig) and complements are also removed during TPE and the patient is potentially posed to infections.^8^ Wing et al. reported increase in opportunistic infections among patients with glomerulonephritis (GN) undergoing TPE. However, most of these patients were receiving immunosuppressive drugs concomitantly.^15^ In a randomized clinical trial on 86 patients with severe lupus nephritis, Pohl et al. showed that TPE did not increase infection risk compared to immunosuppressive drugs.^16^ We found 11 cases of infection, 10 of which occurred in patients with central catheter (jugular or femoral). It is inferred that infection is more related to route of TPE and immunosuppressive drug use than TPE itself.

TPE requires blood flow of about 50-100 ml/min. This can be achieved via large-bore PVC or central (jugular, subclavian, or femoral) catheter. It is assumed that using peripheral access has less complication. Confirming this statement, our study revealed that venous thrombosis, infection, and major complications occurred more commonly in patients with central catheter. Salazar et al. demonstrated that use of ultrasound for inserting peripheral vascular access reduced the need for placement of CVCs.^17^ Therefore, it is a good technique which can obviate the need for central catheter. 

The present study was the first one on TPE complications conducted in Iran. Limitation of our study is its retrospective nature which might have underscored some complications, and causal relationship cannot be proved.

## Conclusion

We found that TPE, as a common treatment of autoimmune disorders, can be associated with major complications in considerable percentage of patients. However, these complications are seen more commonly in patients with CVC. Therefore, use of peripheral vascular access and attentive patient monitoring by trained health providers can reduce its complications.

## Notes:

**How to cite this article:** Afzali M, Oveisgharan S, Rajabkhah S, Abdi S. Complications of therapeutic plasma exchange in patients with neurological disorders. Curr J Neurol 2020; 19(1): 8-12.
